# Gulliver’s virtual travels: active embodiment in extreme body sizes for modulating our body representations

**DOI:** 10.1007/s10339-020-00977-5

**Published:** 2020-06-06

**Authors:** S. Serino, F. Scarpina, A. Chirico, A. Dakanalis, D. Di Lernia, D. Colombo, V. Catallo, E. Pedroli, G. Riva

**Affiliations:** 1 Applied Technology for Neuro-Psychology Lab, Istituto Auxologico Italiano IRCCS, Via Magnasco 2, 20149 Milan, Italy; 2 Department of Psychology, Università Cattolica del Sacro Cuore, Largo Gemelli, 1, 20100 Milan, Italy; 3 Istituto Auxologico Italiano, IRCCS, U.Odi Neurologia e Neuoriabilitazione, Ospedale San Giuseppe, Via Cadorna, 90, 28824 Piancavallo (VCO), Italy; 4 “Rita Levi Montalcini” Department of Neuroscience, University of Turin, Via Cherasco, 15, 10126 Turin, Italy; 5 Department of Brain and Behavioral Sciences, University of Pavia Italy, Piazza Botta, 6, 27100 Pavia, Italy; 6 Department of Medicine and Surgery, University of Milano Bicocca, Via Cadore 48, 20900 Monza, Italy; 7 Department of Basic Psychology, Clinic and Psychobiology, Universitat Jaume I, Av. Sos Baynat, s/n, 12071 Castellón, Spain; 8 Present Address: MySpace Lab, Department of Clinical Neuroscience, University Hospital Lausanne (CHUV), Avenue Pierre Decker 5 CH-1011, Lausanne, Switzerland; 9 Faculty of Psychology, eCampus University, Via Isimbardi, 10, 22060, Novedrate, Italy

**Keywords:** Full-body illusion, Virtual reality, Body representation, Body ownership, Agency

## Abstract

It is noted that the perceptual experience of body and space can be modulated by changing the action capabilities or by manipulating the perceived body dimensions through a multisensory stimulation. This study adds to pre-existing literature by investigating the alterations in bodily experience following embodiment to both enlarged and shrunked bodies, while participants actively navigated in a virtual environment. A normal-sized body served as a reference condition. After each embodied navigation, participants estimated the height and width of three different body parts. Results revealed that the embodiment over shrunked body induced a significant reduction in participants’ body image, while no changes were reported after the embodiment over the enlarged body. Findings were discussed in terms of previous literature exploring the constraints implicated in the ownership over different bodies.

## Introduction

When we interact with the environment, our body perception can change. Traditionally, philosophers, psychologists and neuroscientists make a distinction between “body image” and “body schema” to explain the continuous interaction between the body we perceive and the body with we act. In general terms, the body image includes all conscious perception and emotional feeling about our body, while body schema refers to the unconscious multisensory representations necessary to guide actions in space (De Vignemont 2010; Pitron and De Vignemont 2017). Although we experience our body as relatively stable in our daily life, there is abundant literature suggesting that perception of bodily dimensions can be altered by changing our action capabilities in the environment (Miller et al. 2018; Maravita and Iriki 2004; Longo and Serino 2012). For example, when we are forced to use a stick to reach a far target, we perceive our arm longer, as if the stick has become part of our own body (for a review Martel et al. 2016). Our body judgement can change also when our perception of *body in action* is modified, e.g. when a prosthesis or a wheelchair is used to act in the environment (Ishak et al. 2008; Higuchi et al. 2004; Giummarra et al. 2008; Stefanucci and Geuss 2010). For instance, Ishak et al. (2008) asked participants to judge whether they could reach into an aperture while the size of their hands had been scaled with a prosthesis. Their results indicated that participants adjusted their reachability judgments according to the size of their hands. Such phenomenon can emerge since body representations are highly plastic. This feature allows us not only to integrate a meaningful tool into our body representations (Ishak et al. 2008; Higuchi et al. 2004; Giummarra et al. 2008; Stefanucci and Geuss 2010), but also to embody another fake body part (such as a fake hand) or entire whole body (such as a mannequin or an avatar) through a multisensory stimulation. An increasing number of studies about bodily illusions have found remarkable distortions in bodily perception by experimentally changing the size of the embodied artificial hands and bodies (Normand et al. 2011; Serino et al. 2016; Preston and Ehrsson 2014, 2016; Piryankova et al. 2014; Kilteni et al. 2012, 2015). Using a virtual full-body illusion (i.e. individuals experience the feeling of being the owner of another fake body thanks to the delivery of a synchronous multisensory stimulation on the actual body and its fake virtual counterpart), our group demonstrated that the embodiment over a virtual avatar with a thin body led to a significant shrinkage of subjects’ body representations (Serino et al. 2016; Scarpina et al. 2019).

Interestingly, during an illusionary experience of having a larger or a smaller body, we also perceive the size of the environment consistently with the perceived bodily dimensions (van Der Hoort et al. 2011; Tajadura-Jiménez et al. 2017; van Der Hoort and Ehrsson 2016; Linkenauger et al. 2013; Dijkerman and De Haan 2007). For example, van Der Hoort and colleagues (van Der Hoort et al. 2011) reported that when individuals experienced to be the owner of a “doll body” (i.e. a body of 30 cm), they perceived objects to be larger and farther away. Consistently, when participants were immersed in a “giant body” (400 cm), objects were experienced as smaller and nearer. Thus, our body seems to serve as a *perceptual ruler* we use to measure the apparent size of external objects. Thus, we could re-scale the perceptual world consistently with the perceived dimensions of our body.

Overall, evidence from the experimental studies carried out so far suggested that it is possible to induce temporarily changes in the perceptual experience of the body and surrounding space, by changing the action capabilities in the environment or by manipulating the perceived body dimensions, thanks to a multisensory visuo-motor stimulation.

However, how our bodily experience changes when we are *active* “giant” or “small” agents within a virtual environment, is still an open issue. Using a virtual body in action allows us to investigate the relationship between how we perceive our body and how we act with it. Therefore, this study aimed at making a further step in this field by investigating the alterations in bodily experience following embodiment to both enlarged and shrunked bodies while participants *actively* navigated in a virtual environment. In this sense, virtual reality (VR) represents not only a remarkable tool to study body representations, but it allows to generate in participants the illusion of being and acting in an alternative body, transferring “the sense of what is their own body” to their virtual body representations (Slater et al. 2009), and vice versa, as shown by the studies in which embodiment was induced towards body and body parts with altered physical dimensions (van Der Hoort et al. 2011; Normand et al. 2011; Preston and Ehrsson 2014, 2016; Piryankova et al. 2014; Kilteni et al. 2012, 2015). In our experimental set-up, individuals actively controlled their movements by navigating in a virtual city, while they embodied an enlarged or a shrunked body: they experienced a change in their actions possibilities in the environment resulting from a change in their body dimensions. At the end of each embodied condition, participants reached a commeasured table and they were asked to pick up an object; thus, this ultimate task made the exploratory action goal-directed. Thus, by means of the VR, participants could act and navigate within the environment through a body with a different dimension (i.e. enlarged or shrunken) in comparison with a normal-sized body. In detail, while navigating the virtual environment, participants experienced the illusion to have a body five times smaller (i.e. a height of 34 cm) or larger (i.e. a height of 840 cm) compared with the reference condition. As mentioned, van Der Hoort et al. (2011) showed that it was feasible to induce ownership over various extreme bodies (small, 80 cm; normal, 180 cm; and large, 400 cm). Moreover, Kilteni et al. (2012) found that participants experienced ownership over a virtual arm up to three times the length of the real one and (even less strongly) at four times the length. How is it possible to control an avatar that is extremely different from a “normal-sized body”? Won et al. (2015) coined the concept of “homuncular flexibility” to specifically refer to the control of avatars by using different possibility of actions from those of the physical body repertoire. Specifically, they reported that individuals can efficiently act in a virtual environment through an avatar with three arms.

Our main aim was to test whether this illusionary action-driven experience changed perceptual judgement of bodily dimensions. Thus, at the end of each embodied navigation, participants were asked to estimate the dimension of three different body parts (shoulders, abdomen and hips) and their height (Serino et al. 2016; Scarpina et al. 2019, Keizer et al. 2011).

In line with previous reviewed literature, we expected that body representations would change consistently with the avatars’ dimensions (see Normand et al. 2011; Serino et al. 2016; Preston and Ehrsson 2014; Piryankova et al. 2014; Kilteni et al. 2012). Specifically, the active embodiment over an enlarged body would induce individuals to overestimate their own real bodily dimensions, relatively to an embodiment in a normal-sized body. Conversely, an active embodiment in shrunken would induce the opposite effect of underestimation of body parts size, in comparison with the reference condition.

In order to test whether the illusion was successfully induced, we measured also the level of embodiment through a standard questionnaire, in line with literature about bodily illusions: participants were asked to explicitly report the effects of the illusion as regards *sense of ownership* (i.e. the experience of my body as mine), *sense of agency* (i.e. the feeling of control over my actions) and *self*-*location* (i.e. where I believe where my body is). Since the illusion grounds on a visuo-motor coherence between participants’ movements and virtual actions (Kilteni et al. 2015), we might expect participants to report higher level of embodiment not only in terms of feeling of ownership, but also as concerns the sense of agency, in all experimental conditions (independently from avatar body dimensions).

## Materials and methods

### Participants

A convenience sample of 30 female participants was enrolled via announcements in our university and invited to participate in the study. Participants were eligible to take part in the study if they were female, between 18 and 55 years of age, had no history of neurological diseases, no current physical conditions (pregnancy) known to influence their body size, and if they reported not to have a current or prior history of psychiatric illness. In addition, participants were required to have a body mass index (BMI) between 18.5 and 25 kg/m^2^, with a height ≥ 160 cm since our “normal-sized body” was 170 cm tall. During the screening for eligibility, two participants were excluded since they were under- or overweight, and two other participants were excluded because they are not enough tall (154 cm). Thus, 26 eligible participants took part in the study [mean age of 24.19 (SD = 3.19), mean BMI of 20.22 (SD = 1.27)].

There was no significant difference between the height of the normal-sized body avatar and the mean height of our sample [mean = 167.546 (SD = 6.574); *t*(25) = − 1.903; *p *= 0.069].

The experiment was conducted in compliance with the Helsinki Declaration (of 1975, as revised in 2008), and it was approved by the Ethical Local Board of the “Università Cattolica del Sacro Cuore” (Catholic University of the Sacred Heart, Milan, Italy).

### Virtual reality (VR)-based full-body illusion

The experimental apparatus consisted of a head-mounted display (HMD, Oculus Rift DK2), a hand-tracking device (Leap Motion). The application was developed with the software Unity3D (www.unity3d.com). The Leap Motion sensor was placed on the front of the Oculus Rift using the Orion beta SDK for VR support to replicate the participants’ hands movements in real time allowing a synchronous visuo-motor coherence between actual and virtual actions. Leap motion is a hand motion-sensing device to track hand movements in virtual reality, consisting of two cameras and three infrared LEDs. In order to move the avatar forward, participants were asked to raise their hands in front of their face with palm direction straight ahead. The experiment was running on a Workstation HP Z620 with processor Xeon E5 2660 V2 2.2 GHz 25 MB Cache, 64 MB of RAM and Nvidia K6000 graphic card with 12 GB of dedicated RAM.

The application featured a virtual city with several buildings, shops and cars. Crucially, people’ body perception may be affected by serial dependence bias (i.e. a tendency to perceive a current stimulus more like previous ones) (see Alexi et al. 2018). In other words, judgments of their own body size could be biased towards a previously viewed body. Therefore, no human characters were present within the virtual environment.

Participants’ hands were shown in the environment as realistic-like virtual models to be used to for navigation. As previously explained, our set-up included combination of Oculus Rift (HMD device) and Leap Motion (hand-tracking device) for first-person movement control in virtual environments. To control avatars movements, participants were invited to raise their hands in front of their face with palm in straight-ahead direction. Participants could turn their head freely to change their point of view thanks to the head sensor trackers provided by Oculus Rift.

**Fig. 1:**
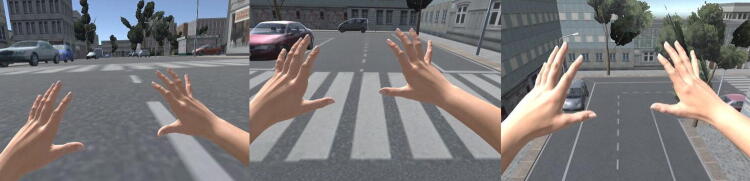
Virtual reality (VR)-based full-body illusion. Participants were asked to stand upright and to position their hands forward to actively navigate in the virtual environment. They were instructed to follow a predefined route of about 90 s. The participants’ hands were represented in the environment as realistic-like virtual models to be used for navigation. The congruence between the participants’ hands movements and avatar ones allowed participants to feel ownership over virtual bodies. Three different experimental conditions: “Active embodiment in a shrunked body” (left part), “Active embodiment in a normal-sized body” and “Active embodiment in an enlarged body” (right part)

All participants were exposed to three different experimental conditions (Fig. 1):

- “*Active embodiment in a normal*-*sized body*”, namely an avatar who is 170 cm tall;
- “*Active embodiment in an enlarged body*”, namely an avatar who is 850 cm tall;
- “*Active embodiment in a shrunked body*”, namely an avatar who is 34 cm tall.

**Fig. 2:**
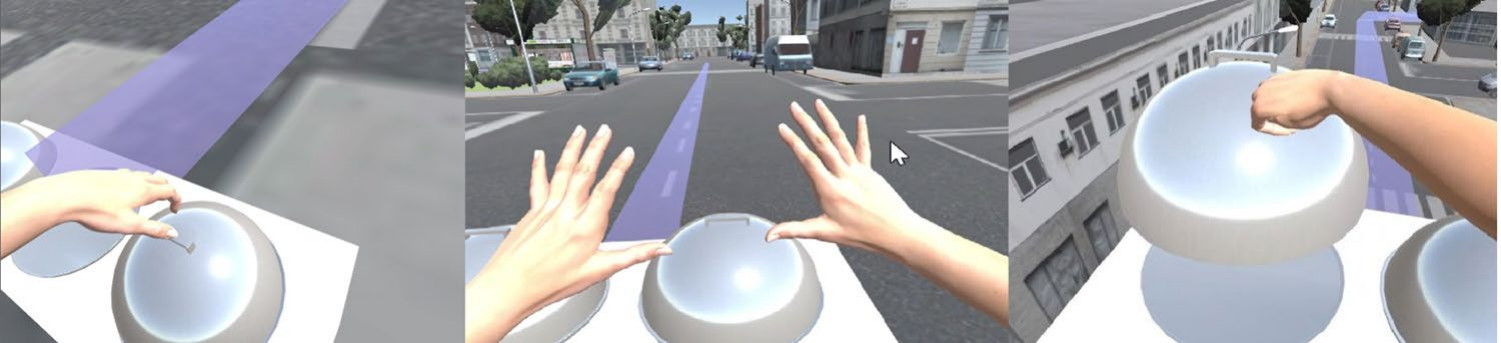
Participants were asked to follow a predefined route of 90 s (i.e. indicated by a red line) in the virtual city to reach a proportionally sized table: “Active embodiment in a shrunked body” (left part), “Active embodiment in a normal-sized body” and “Active embodiment in an enlarged body” (right part)

In each condition, the size of virtual hands (i.e. the only visible body part of the avatar) was commeasured to the avatar’s height. Participants were asked to follow a predefined route of 90 s (i.e. indicated by a red line) in the virtual city to reach a commeasured table and, there, to pick up an object. This action marked the end of each embodied condition (Fig. 2). To keep the duration of each experimental condition equal (i.e. 90 s), we manipulated the walking speed according to the changes in body dimensions. This way, we also provided participants with a more embodied experience:

- “*Active embodiment in a normal*-*sized body*”: 3 m/s;
- “*Active embodiment in an enlarged body*”: 4.5 m/s;
- “*Active embodiment in a shrunked body*”: 0.7 m/s.

**Fig. 3:**
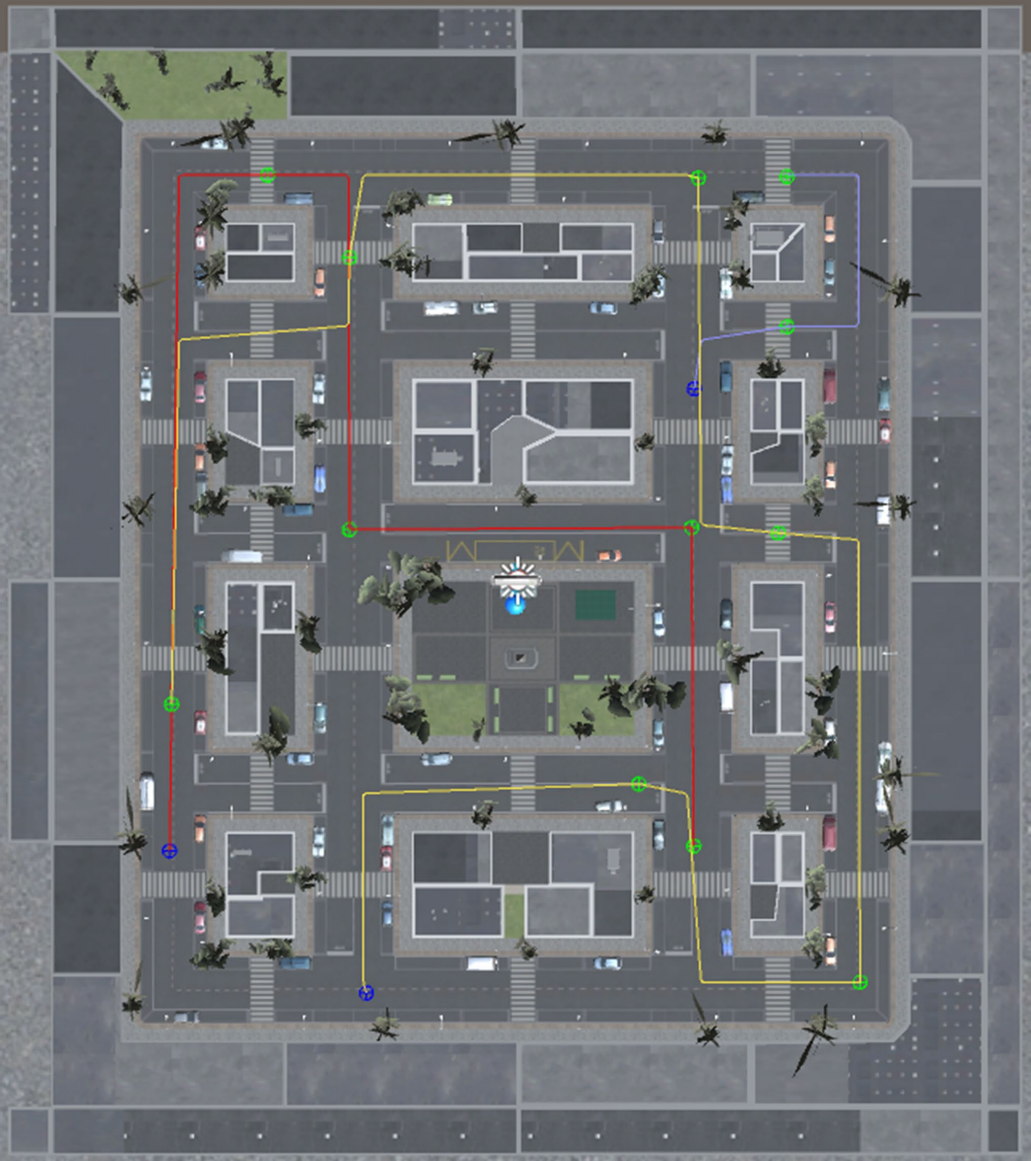
Length of route (from starting to ending point): “Active embodiment in a normal-sized body”: 275 m (red line), “Active embodiment in an enlarged body”: 420 m (yellow line) and “Active Embodiment in a shrunked body”: 63 m (blue line)

In Fig. 3, routes for each embodied condition are depicted.

Considering that the ratio between a person’s height and stride distance is about 0.4, participants in all experiment conditions had a virtual physical effort of < 400 virtual steps in 90 s.

### Procedure

At the arrival in the laboratory, participants received written information about the study and were asked to sign the informed consent form to participate in the study. All participants were weighed and underwent a brief interview to ensure that they met the study criteria. Subsequently, all participants were invited to wear the HMD to perform the three different experimental conditions planned. The interpupillary distance was individually calibrated at the start of the experiment. The first experimental condition (“Active Embodiment in a normal-sized body”) was maintained as the first one for all participants, while the other two experimental conditions were within-participants randomized. After each virtual experience, all participants were asked to complete the body size estimation task (Serino et al. 2016; Scarpina et al. 2019, Keizer et al. 2011) and to complete the adapted version of the Embodiment Questionnaire (Serino et al. 2016; Scarpina et al. 2019).

### Measurements

#### Body size estimation task

To investigate whether the embodiment in different active extreme bodies was able to induce changes in body representations, a body size estimation task was employed after each experimental condition (“Active embodiment in a normal-sized body”, “Active embodiment in an enlarged body” and “Active embodiment in a shrunked body”). For each estimation, participants were asked to stand about 3.5 metres from a whiteboard equipped with a red vertical line as reference. They were provided with a laser beam to make the body part estimations on the whiteboard. First, participants were asked to estimate their own height. After, participants estimated the width of three different body parts (i.e. shoulders, abdomen and hips) by using the same laser beam. These estimates were made in a random order within participants. Actual body part dimensions were measured by the experimenter at the end of the experiment, to avoid any bias in the subjective judgment.

#### Embodiment questionnaire

At the end of each experimental conditions, an adapted version of the Embodiment Questionnaire (Serino et al. 2016) was administered. The self-report questionnaire was composed of 15 items on a seven-point Likert scale. This instrument assessed how participants experienced the illusion on three different dimensions: *body ownership* over the virtual body (11 items, total score: 77), *self*-*location* (2 items, total score: 14) and *agency* (2 items, total score 14). The score for each sub-scale was calculated as the sum of items.

Two additional questions were added to check whether individuals would perceive to self-locate themselves in the virtual city and use their body as metric for space perception: 1) “I felt as the virtual city was immensely bigger than me” (seven-point Likert scale) and 2) “I felt as the virtual city was immensely smaller than me” (seven-point Liker scale).

### Data analysis

As first step, the percentage of misestimation for each body part was calculated as follows:

$${\text{percentage }}\,{\text{of}}\,{\text{ misestimation }} = \, \left( {{\text{estimated }}\,{\text{size}}{-}{\text{actual }}\,{\text{size}}} \right)/{\text{actual}}\,{\text{ size}}) \, *100$$

according to Keizer et al. (2016). Specifically, a negative value represents an underestimation, while a positive value represents an overestimation. Prior to analyses, normality of data distribution was checked. To investigate changes in body representations between experimental conditions, a repeated measure ANOVA with *Condition* (“active embodiment in a normal-sized body” vs. “active embodiment in an enlarged body” vs. “active embodiment in a shrunked body”) as within-subject variable was separately conducted for each body part. Bonferroni’s adjusted post hoc comparison *t* tests were computed to break down significant effects. Eventually, to evaluate potential differences in embodiment experience across the three experimental conditions, a series of repeated measure ANOVAs with *Condition* as within-subject variable were separately carried out on the three sub-scales of the Embodiment Questionnaire (i.e. ownership, self-location and agency). Also, in this case, Bonferroni’s adjusted post hoc comparison *t* tests were calculated to break down significant effects. Two paired-sample *t* tests were carried out to investigate potential effects on two single items on virtual space perception.

All these statistical analyses were conducted using the Statistical Package for the Social Sciences for Windows (SPSS Inc., Chicago, IL, USA), version 23.

## Results

### Body size estimation task: height

No significant effect in height perception emerged after embodying different bodies of extremely different sizes [*F*(1.538; 38.459) = 2.565; *p *= .102; Partial *η*^*2*^ = .093). Participants reported the same level of accuracy in estimating their height, regardless of conditions [M_normal-sized body_ = − 1.902 (SD_normal-sized body_ = 3.435); M_enlarged body_ = − .911 (SD_enlarged body_ = 3.749); M_shrunked body_ = − 2.588; SD_shrunked body_ = 3.524).

### Body size estimation task: body parts

**Fig. 4:**
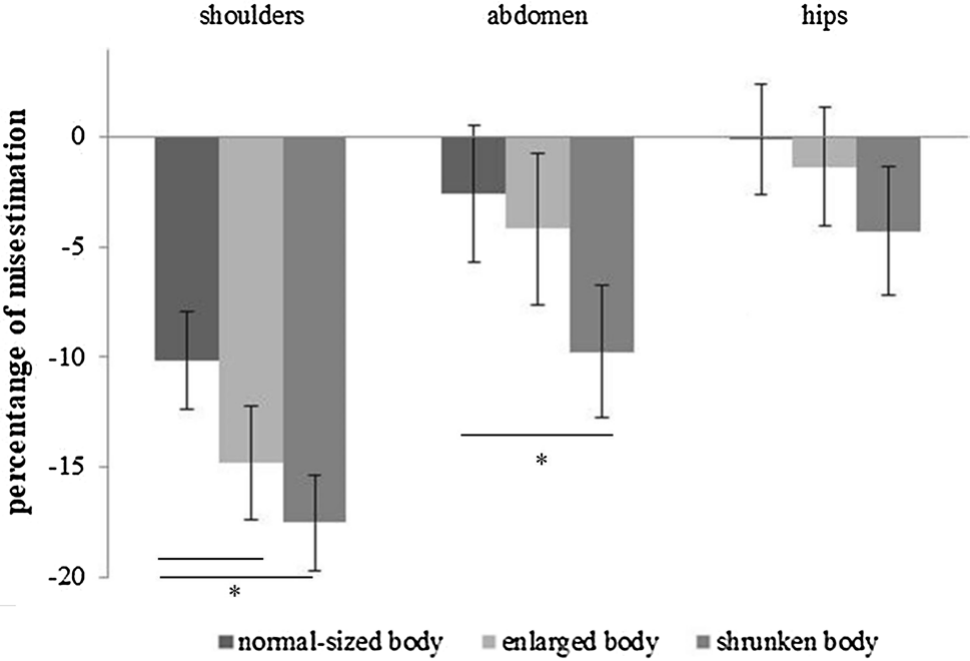
Body parts estimation task. Changes in width estimations after the exposure to the experimental conditions. *Represents significant differences between conditions, according to post hoc comparisons

Results from a series of repeated measure ANOVAs with Condition (“Active embodiment in a normal-sized body” vs. “Active embodiment in an enlarged body” vs. “active embodiment in a shrunked body”) as within-subject variable conducted for each body parts are presented in Table 1. Bonferroni’s adjusted post hoc comparison *t* test results are presented in the right side of the table and shown in Fig. 4.

**Table 1:** Body part estimation task

	Mean (SD)	*F*^b^	*p*	Partial *η*^2^		Active embodiment in an enlarged body	Active embodiment in a shrunked body
Shoulders							
Active embodiment in a normal-sized body	− 10.165 (12.208)	11.572	<.001	.316	Active embodiment in a normal-sized body	*t*(25) = 3.321; *p *= .003, *d* = .354, 95% CI [1.77, 7.54]	*t*(25) = 4.303; *p *< .001, *d* = .594, 95% CI [3.75, 10.63]
Active embodiment in an enlarged body	− 14.818 (14.016)				Active embodiment in an enlarged body		*t*(25) = 1734; *p *= 0.095, *d* = .194, 95% CI [− 1.48, 5.55]
Active embodiment in a shrunken small body	− 17.353 (11.991)						
Abdomen							
Active embodiment in a normal-sized body	− 2.858 (17.075)	3.547	.036	.124	Active embodiment in a normal-sized body	*t*(25) = .536; *p *= .597, *d* = .113 95% CI [− 3.81, 6.48]	*t*(25) = 2.641; *p *= .014, *d* = .410 95% CI [− 1.52, 12.30]
Active embodiment in an enlarged body	− 4.196 (18.978)				Active embodiment in an enlarged body		*t*(25) = − 1. 795; *p *= .085¸ *d* = .272 95% CI [− 0.82, 11.97]
Active embodiment in a shrunken small body	− 9.770 (16.526)						
Hips							
Active embodiment in a normal-sized body	− 095 (13.084)	3.694	.032	.129	Active embodiment in a normal-sized body	*t*(25) = .892; *p *= .381, *d* = .091 CI [− 1.92, 4.85]	*t*(25) = 2.361; *p *= .026, *d* = .288 95% CI [− 0.56, 8.21]
Active embodiment in an enlarged body	− 1.371 (14.743)				Active embodiment in an enlarged body		*t*(25) = − 2.097; *p *= .046, *d* = .190 95% CI [0.05, 5.78]
Active embodiment in a shrunked body	− 4.288 (15.924)						

Results from a series of repeated measure ANOVAs with *Condition* (“active embodiment in a normal-sized body” vs. “active embodiment in an enlarged body” vs. “active embodiment in a shrunked body”) as within-subject variable conducted for each body parts

Bonferroni’s adjusted post hoc comparison *t* test results are presented in the right side of the table

^a^Data are shown as mean (SD)

^b^For all analyses, *df* = 2.50

Referring to the shoulders’ width estimation, a significant effect of *Condition* emerged. Participants underestimated the width of their shoulders after embodying both an enlarged and a shrunked body with respect to the normal-sized body, but without a difference between these two conditions. With concern to the estimations of the abdomen, findings indicated a significant difference between the three embodied conditions. Specifically, participants underestimated abdomen perception following embodiment in a shrunked body when compared to the normal one, where they were quite accurate (Table 1). No other comparisons were significant. Regarding hips’ width estimation, the embodiment in different body sizes resulted in significant changes in body perception. Specifically, there was a marginally significant increase in the underestimation tendency after the embodiment in the shrunked body with respect to the embodiment in a normal-sized body, where participants were quite accurate in their estimates but it did not reach statistical significance after Bonferroni adjustment. No other comparisons resulted significant.

### Embodiment questionnaire

**Fig. 5:**
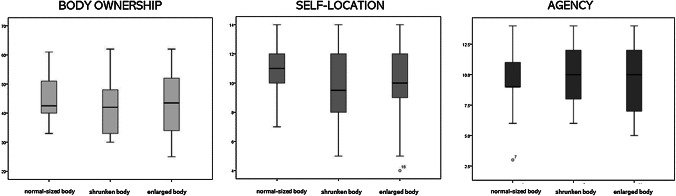
Results obtained from the Embodiment Questionnaire. Feeling of embodiment over virtual bodies after the exposure to the three experimental conditions (i.e. “Active embodiment in a shrunked body”, “Active embodiment in a normal-sized body” and “Active embodiment in an enlarged body)

Concerning the scores relative to the component of *body ownership* and the component of *agency*, no significant difference was found between the three experimental conditions. A main effect of *Condition* emerged for s*elf*-*location* score [*F*(2,50) = 6.833; *p *= .002; Partial *η*^*2*^.215], with a higher score for the normal-sized body condition compared to embodiment over the avatar of extreme body sizes [active embodiment in a normal-sized body–active embodiment in an enlarged body: *t*(25) = 2.692; *p *= .012, *d* = .562; active embodiment in a normal-sized body–active embodiment in a shrunked body: *t*(25) = 3.467; *p *= .002, *d* = .511]; instead, no difference emerged when embodiment in both extreme-sized bodies was compared [*t*(25) = − .340; *p *= .736, *d* = .045] (Fig. 5).

Focusing on the two single items relative to the virtual space perception, participants experienced the virtual city bigger when immersed in an shrunked body, compared to the enlarged condition [*M*_shrunked body_ = 5.346; SD_shrunked body y_ = 2.096); *M*_enlarged body_ = 2.692; SD_enlargedbody_ = 2.223; *t*(25) = 4.059; *p *= <.001; *d* = 1.228]. In the same way, when participants embodied the larger body, they perceived the space as smaller compared to when they embodied the shrunked body [*M*_shrunked body_ = 1.769; SD_shrunked body_ = 1.423); *M*_enlarged body_ = 4.577; SD_enlarged body_ = 2.301; *t*(25) = − 4.308; *p *= <.001; *d* = 1.467].

## Discussion

The aim of the present experiment was to investigate changes in body representations after an action-driven embodiment over a body with extreme (enlarged or shrunken) dimensions, when compared to the embodiment towards a normal-sized body.

In line with our hypothesis, we found that after having experienced a shrunked body, participants reported a significant underestimation of their body size; in different words, participants represented their bodily dimensions as shorter after they had embodied a shrunked body. Instead, in the case of enlarged body, we found a significant change only about shoulders, but it was in the opposite direction of our a priori hypothesis. Participants underestimated (and not overestimated) the horizontal dimensions of their shoulders, when they had experienced a sense of ownership towards a larger body.

Our results were partially in contrast to some previous findings indicating that embodying a different body or body parts should result in congruent effects on body size estimates (e.g. Kilteni et al. 2015). However, some inconsistencies can be found in previous literature about how experimental manipulation is effective in manipulating the perceived body size. For instance, in the Rubber Hand Illusion experiment described by Haggard and Jundi (2009), participants experienced the illusion of ownership towards the fake hand, while watching a tactile stimulation provided with a large or small glove. When inviting to grasp cylinders of identical size, but different weights, only the embodiment with larger gloves influenced participants’ action, since they perceived the cylinders as heavier. Instead, no change in body perception after embodying small gloves was observed. In another study employing this bodily illusion (Pavani and Zampini 2007), in which the dimension of the fake hand was manipulated, it was reported that the illusion of ownership can be efficiently induced towards a rubber hand with enlarged dimensions, as well as with veridical dimensions, but not towards a shrunken rubber hand. The authors interpreted these results adopting a “top-down perspective” of body ownership: individuals more likely accommodated the bigger or veridical hands in their pre-existing body representations. Indeed, it is well known that some experimental constraints about spatial–temporal sensory congruency should be *sine qua non* to induce a successful embodiment towards body parts or full body (De Vignemont and Farnè 2010; Tsakiris 2010; Costantini and Haggard 2007). For instance, in the Rubber Hand Illusion, the strength of the illusion seems to decrease when a non-corporeal object (such as a wooden stick) was used or when rubber hand’s posture is spatially incongruent with respect to the real hand (e.g. Haans et al. 2008; Tsakiris and Haggard 2005; Guterstam et al. 2013; Costantini and Haggard 2007). These results supported the “Body Model Hypothesis” (De Vignemont and Farnè 2010), according to which a “long-term body image” (O’Shaughnessy 2008), or a “body structural description” (Schwoebel and Coslett 2005)), or a “body memory” (Riva 2018)—i.e. how we perceive our body or body parts—plays a critical role in determining what can be efficiently embodied. We embody efficiently in our body representation only objects and external tools that match anatomically, spatially, temporally and/or functionally our pre-existing body representations. Aymerich-Franch and Ganesh (2016) recently introduced the hypothesis that the top-down regulation of embodiment over an external body is regulated also by the functionality of limbs, namely as opportunities of actions for the stimulated limb (following the Gibson’s idea of affordances). For instance, it would be more difficult to embody a wooden stick (Tsakiris and Haggard 2005), beyond anatomical difference between pre-existing body model, if the stick is not used for goal-directed action, such as grasping movements. Considering this “functional model” of ownership, we might assume that our participants accommodated the shrunked body into their pre-existing body model more efficiently in comparison with the enlarged one, since they believed that the active navigation might be possible in the case of a smaller body, but not in the case of a larger one: potentially, the larger body might be physically heavier or dangerous because of falls.

Another line of interpretation of our results might be offered by the so-called co-construction model about the reciprocal relationship between body image and body schema (Pitron and De Vignemont 2017). According to this model, there might be a mutual influence between these two kinds of cognitive representations; *anticipatory models* based upon previous body representations (i.e. priors) fundamentally sustain the creation of different body raw representations together with common multisensory inputs, that nonetheless can interact each other through multisensory integration processes, effectively reshaping their own features. Therefore, the representations based upon perceptions (i.e. body image) can partially alter the representations based upon action (i.e. body schema), and vice versa. Further exploring this perspective, it is therefore possible to suppose that body representations based upon information relevant for action (i.e. “raw body scheme”) can act as functional constraints in modulating the bodily experience. Common anticipatory models (i.e. priors), which represent core and stable features, could be resiliently utilized across different modality as representations anchoring the subject to functional (for action) representation. In this perspective, we might also assume that it could be easier for an individual to experience a smaller body (e.g. curling up) than live the same experience in larger one. Some physical priors can probably be considered as structural and therefore very hard to change.

Finally, we might look at our results embracing a psychological perspective. It is well known that in Western societies, enlarged bodies (i.e. characterized by larger physical dimensions) are socially not desirable and stigmatized (Puhl and Heuer 2009).

Preston and colleagues investigated the relationship between body perception and body satisfaction using full-body ownership illusions. In a series of studies, they found the illusory ownership over a slimmer mannequin body led participants in perceiving their actual body as slimmer and reporting higher feelings of body satisfaction (Preston and Ehrsson 2014); whereas ownership over an obese virtual body reduced body satisfaction (Preston and Ehrsson 2016). In our case, feelings of body dissatisfaction could have played a role after embodying an extreme large body.

Regarding the counterintuitive enhancement of underestimation after the embodiment in the bigger body, it is worthy to mention that there was a tendency towards the underestimation of shoulders after all embodied conditions (see Table 1). Longo and colleagues (Sadibolova et al. 2019) confirmed the presence of large body size distortions also in normal-weight population, and interestingly, they emphasized that the largest underestimation was found for the volume of the torso. More recently, we reported that healthy participants perceived their shoulders larger after they have embodied a skinny avatar (Scarpina et al. 2019). These opposite results might suggest that bodily illusions affect body parts representations differently; however, it still an open question which body parts and in what circumstances can be misperceived, and in which direction.

Regarding the Embodiment Questionnaire (Serino et al. 2016; Scarpina et al. 2019), participants reported to have experienced a solid illusion in terms of body ownership and agency, independently from the experimental condition. Participants perceived the avatar as their own actual body, as well as the avatars’ movements as their own movements, despite its size. Instead, the feeling of being in specific location in the space, i.e. the s*elf*-*location*, increased only after the embodiment over the normal-sized body. This result was not a priori expected. We did not expect changes in the perceived self-location between the three different embodiment conditions: in our VR illusionary experience, we did not any conflict between the spatial location of the actual and the virtual body, as instead it is generally done in the experimental induction of the out-of-body experience (for a review of the distinct mechanisms and neural correlates underlying body ownership and self-location, please see Serino et al. 2013). Indeed, mean responses from our participants in all the experimental conditions were quite high (Fig. 4), suggesting the absence of a “true” conflict in the perceived location between the two bodies. As in Maselli and Slater (2014), our slightly higher scores in normal-sized condition in self-location can mirror slightly higher scores for the same experimental condition on ownership sub-scale. Thus, the sense of ownership could work as a “driver” for self-locate themselves in the virtual scene. However, we cannot exclude that this interesting, even though unexpected result, might be due to the experimental procedure, according to which our participants tested normal-sized body always before the other two conditions.

Finally, in all embodied conditions we observed that participants reported a higher agency score (i.e. feeling of being in control over bodily actions), resulting from a matching between intentions to act with the results of such own actions (active conditions) (Sato and Yasuda 2005; Kalckert and Ehrsson 2012; Tsakiris et al. 2010). These findings are in line with studies suggesting that agency—in our study enhanced by the congruent response of the environment during the interaction—has a relevant role in eliciting the illusion of being the owner of another body (Kalckert and Ehrsson 2012).

Regarding the space perception, our participants subjectively perceived the size of external environment congruently with the size of their owned body. This finding is in line with previous literature that indicated an effect of embodying bodies of different sizes on the perceptual experience of space (van Der Hoort and Ehrsson 2016; van Der Hoort et al. 2011; Tajadura-Jiménez et al. 2017). One renowned explanation for the rescaling of perceived space has roots in Gibson (1966)’s theory of affordances: world might be thought as full of “opportunities for action”. With this regard, individuals would perceive the external environment (and therefore rescale the external environment) not only in terms of actions, but on the basis of their intention to act (see Witt et al. 2005).

Some limitations should be noted in our study. First, we did not report any body size estimation before the illusion, as in Serino et al. (2016). Since participants may gradually improve (practice) or decline (fatigue) in their body estimations, in this work we opted to avoid a baseline body size estimation task before the virtual reality experience. Accordingly, we decided to have this condition always as first for all participants to use it as a reference measurement according to which we performed the comparisons against the other extreme conditions, instead of performing a baseline measurement outside VR. However, it should be taken into account that healthy individuals generally reported large distortions in body part/whole-body estimation (Sadibolova et al. 2019). Second, concerning the sample, in this study we did not screen participants for body perception concerns or eating disorders symptoms, as in our previous study (Serino et al. 2016). However, the presence of subclinical symptoms of eating disorders may modulate changes in bodily experience following a bodily illusion (Preston and Ehrsson 2014). Third, measuring changes in space perception after each embodiment conditions (and not only the space perception in general) could provide further evidence about the link between body and space in terms of “affordances”. Finally, the sample included only female participants. Gender is one of the most influencing factors for both body perception and navigation skills. It is well known that body dissatisfaction experience of men, including specific body areas of concern, is qualitatively different from that of women. For example, males expressed less concerns about their body image or drive for thinness, but they showed higher concerns with muscularity and body shape (Dakanalis et al. 2015, 2016a, b). Gender differences should characterize also the navigation experience; for example, a recent meta-analysis confirmed that the male participants outperform female participants, with a small to medium effect size (Nazareth et al. 2019). Thus, our findings may not be fully generalizable to the entire population. Another potential confounding effect might be read in the different speed for the three tested conditions. In order to enhance embodiment towards the avatar, we adjusted in particular the speed of shrunken condition. According to preliminary pilot studies, and specifically to what reported by naïve participants, when we kept the velocity constantly between conditions, individuals had some difficulties to embody the avatar. In particular, when participants were asked to embody a small body at higher speed, they reported a weird feeling of “running” or “being embodied in a mouse”, instead of walking However, since our consideration was based on only qualitative feedbacks from small pilots, future research should address this topic and to verify whether changing the relationship speed-body mass might increase or decrease body ownership. Moreover, future studies are needed to expand our knowledge on active embodiment and its influence on body and space perception, e.g. including the use of a real-time motion capture to track and reproduce the entire participants’ bodies in virtual environments (and not only participants’ arms). In this way, a comparison between a synchronous condition (i.e. avatar moving in synchronous with participants’ movements) and an asynchronous one (i.e. avatar moving with a delay in respect to participants’ movement) would allow us to deepen the relationship between ownership and agency on body perception.

Despite these limitations, our results supported the “functional model” of body ownership and its constraints (Aymerich-Franch and Ganesh 2016); moreover, it expanded the literature about the complex processes through which people can incorporate other body parts into their own body.

## Data Availability

All data that support the findings of this study are available on request from the corresponding author.
